# Blockchain and IoT Integration: A Systematic Survey

**DOI:** 10.3390/s18082575

**Published:** 2018-08-06

**Authors:** Alfonso Panarello, Nachiket Tapas, Giovanni Merlino, Francesco Longo, Antonio Puliafito

**Affiliations:** Department of Engineering, Universita’ degli Studi di Messina, Contrada di Dio, S. Agata, 98166 Messina, Italy; ntapas@unime.it (N.T.); gmerlino@unime.it (G.M.); flongo@unime.it (F.L.); apuliafito@unime.it (A.P.)

**Keywords:** Internet of Things, IoT, blockchain, survey, machine economy

## Abstract

The Internet of Things (IoT) refers to the interconnection of smart devices to collect data and make intelligent decisions. However, a lack of intrinsic security measures makes IoT vulnerable to privacy and security threats. With its “security by design,” Blockchain (BC) can help in addressing major security requirements in IoT. BC capabilities like immutability, transparency, auditability, data encryption and operational resilience can help solve most architectural shortcomings of IoT. This article presents a comprehensive survey on BC and IoT integration. The objective of this paper is to analyze the current research trends on the usage of BC-related approaches and technologies in an IoT context. This paper presents the following novelties, with respect to related work: (i) it covers different application domains, organizing the available literature according to this categorization, (ii) it introduces two usage patterns, i.e., device manipulation and data management (open marketplace solution), and (iii) it reports on the development level of some of the presented solutions. We also analyze the main challenges faced by the research community in the smooth integration of BC and IoT, and point out the main open issues and future research directions. Last but not least, we also present a survey about novel uses of BC in the machine economy.

## 1. Introduction

Internet of Things (IoT) is a ubiquitous internet work of intelligent physical objects, called “Things,” and people. IoT empowers any“Thing” to connect and communicate, thereby, converting the physical world into an enormous information system. Various technologies, like Cloud Computing and Machine Learning to Data Analysis and Information Modeling, are quickly becoming an integral part of IoT fabric. The tremendous advancement in the field of IoT is causing growth in Information and Communication Technology (ICT) business as well. IoT is enabling the development of new business methods, and one of its most essential aspects resides in the data enhancement that will affect the growth in the ICT market. The extent to which IoT will be part of our day-to-day life can be understood from the fact that 95% of newly introduced products by 2020 will have IoT technology at its core [1].

With an ever-increasing presence of IoT objects and their visibility from the Internet, security, i.e., the legitimate users access to resources, is of prime concern. On the one hand, the ubiquitous nature of IoT encourages the creation of innovative applications for the end user, but, on the other hand, lack of security measure may lead to critical issues like persons subjected to physical damage such as burglary due to the hacking of the smart alarm system. Security has another aspect, namely “*the privacy concern*,” associated with it. Centralized companies managing sensitive user data can use them illegitimately, thus leading to a breach of privacy [2]. Aggravating the situation is the fact that some years ago to think about a scenario with billions of connected devices was quite unlikely and, for this reason, the security aspects have not always been considered at the design phase of the products. In fact, as per studies conducted worldwide by Gartner, IoT security spending will reach $1.5 billion in 2018, and, by 2022, half of all security budgets for IoT will go to fault remediation, recalls and safety failures rather than protection [1]. Therefore, the progressive expansion of the business related to this type of always connected environments implies new technological challenges and implications about security, privacy, and interoperability.

A distributed trust technology, ensuring scalability, privacy, and reliability, is a cornerstone for the growth of such IoT environments. In recent years, the Blockchain (BC) technology has matured significantly and is seen as a promising solution in achieving the goals mentioned above thanks to its intrinsic security. In fact, BC is a “secure by design” system that can mitigate security risks owing to its capabilities such as immutability, transparency, auditability, data encryption and operational resilience.

### Key Contribution

In recent years, researchers have been trying to address the problem of integrating BC with IoT [3,4]. Reyna et al. [5] analyzed the challenges emerging from the integration of IoT and BC. They presented possible ways of integration and platforms that are integrating IoT and BC in a general context. In contrast to the above work, we present an extensive survey categorized by application areas like the smart city. We try to give another perspective to the survey, focusing on specific use-case and goals, specifying the “technologies” or “models” used instead of considering the operating environment of the solution. We also present a unique aspect of BC in the machine economy, enabling data marketplaces. Kouicem et al. [6] presented a general survey of security challenges in IoT with BC being one of the solutions. Similarly, Jesus et al. [7] surveyed the application of BC to secure IoT and presented “Stalker” attack. Opposed to the above surveys, we present a focused survey, highlighting the suitability of IoT and BC integration. In addition to BC addressing the security challenges, we also present the innovative solutions emerging from the integration.

Atzori et al. [8] critically reviewed BC-based platforms for IoT and highlighted the limitations of applying BC in the IoT environment. Compared to it, we present recent developments in the field of IoT and BC integration. We also present an extensive survey of solutions emerged in recent years. Christidis et al. [9] provided a taxonomy about the BC typologies evaluating pros and cons of introducing BC into IoT. Several ideas have been proposed such as the use of BC and InterPlanetary File System (IPFS) to update the firmware of the IoT devices by activation of smart contracts or the set-up of an environment where devices can gain “money” by selling or buying resources (TransActive Grids [10]) or data (FileCoins [11]). In contrast to the above work, we present a taxonomy based on application to IoT environment and present an exhaustive survey in each of those categories. We also try to motivate the user by presenting the innovative application of BC in the IoT domain.

Conoscenti et al. [12] analyzed literature extensively about the possible applications of BC, presenting its different forms to solve some of the security challenges. In contrast to the limited scope of this work where only four papers dealing with IoT and BC integration were discussed, we present a more exhaustive survey of BC-IoT integration. Table 1 summarizes the most relevant ones and categorizes them by contributions, also highlighting where this paper places itself.

In contrast to work presented in [5,6,7,8,9,12], this paper focuses on the presentation and categorization of several solutions for the application of BC in an IoT environment. This article presents the following novelties concerning the related works:

- It offers different application areas, organizing the available literature in these fields.
- It presents two usage patterns that are: device manipulation [13] and data management (open marketplace solution).
- It discusses the development level of some of the offered solutions.

The rest of the paper is organized as follows. In Section 2, we present the motivation behind BC and IoT integration. Section 3 presents the principle characteristics of BC. The following Section 4 is the core of the paper. Here, a literature review of the integration between IoT and BC is provided. Section 5 discusses given solutions available in IoT environments. In Section 7, we analyze the challenges in the integration of BC and IoT, and challenges resulting from the integration. Section 6 provides a comparison among the BC-based marketplaces available in the literature, and, finally, Section 8 concludes our work and outlines future research perspectives.

## 2. Motivation: Need for Blockchain

Today, IoT accounts for 5 billion connected devices, and this number will continue to grow and reach 29 billion by 2022 [14]. Every device produces and exchanges data on the Internet. Thus, considering these massive number of devices, it is easy to understand that we are talking about an extensive and continuous production of data. Addressing the fundamental security issues for such a vast information system is a challenge in itself. In this section, we discuss the challenges faced by IoT deployments.

An essential challenge for IoT is its distributed architecture. Typically, in an IoT network, each node is a possible point of failure that can be exploited to launch cyber attacks such as Distributed Denial-of-Service (DDoS) [15]. A system of nodes with several infected devices, acting simultaneously, can collapse quickly. Another concern regards its centralized configuration (typically IoT environment leverages on a central cloud service provider) [16]. Such a central point of failure is a vulnerability, which must be addressed. Another constant and probably one of the most critical threat is data confidentiality and authentication [17]. In the absence of data security, IoT data can be exploited and inappropriately used. In addition, with the emergence of new business models where devices can exchange resources like data, computational power or electricity autonomously, data security becomes critical.

Another challenge for IoT is data integrity [16]. One of the significant applications of IoT is in decision support systems. The data aggregated from the fleet of sensors can be utilized in making timely decisions. Thus, it is essential to protect the system from injection attacks, which try to inject false measures and therefore, affect decision-making. Availability is critical for automated systems like vehicular networks, manufacturing industries, and smart grids which are processing real-time information [16]. Sensor downtime can result in losses varying from monetary to life-threatening situations. With the emergence of Machine Economy, whereby the sensors generating data are capable of trading data in data marketplaces and end-to-end autonomous system, creating trust between participating entities is a significant challenge [18]. The presence of a publicly verifiable audit trail without a trusted 3rd party is desirable, thus solving the problem of non-repudiation.

## 3. Blockchain Principles and Strengths

The primary goal of the BC is to free people from any form of trust we are now forced to give to intermediaries who regulate and “manage” a large part of citizens’ life. The BC is a technology that initially was used to promote commercial transactions (trades) through a new currency that is independent of banks and States, called Bitcoin [19]. This currency is digital, and it is used as a means of exchange accepted by the users involved in a transaction. The strength of this currency or more specifically cryptocurrency is that there is no need for a public authority. However, what is much more interesting is to figure out how to realize and implement this new cryptocurrency.

To achieve such a goal, several technologies, and security and cryptographic functions have been exploited. The synergy among these all technologies constitutes the BC. However, this technology is starting to be exploited in several different contexts and not just for Bitcoin. This is the most interesting point of this revolutionary new technology. People often confuse BC with Bitcoin, but Bitcoin indicates a cryptocurrency that leverages the BC technology to be able to freely and globally circulate without the supervision of a central guarantor (the banks). In other words, Bitcoin is only a financial use case that makes use of this powerful technology.

Before we dive into the BC technology and figure out what BC attempts to solve, it is important to make an important precondition. The BC is merely a distributed database system based on consensus rules that allow the transfer of value between entities. There are many distributed systems based on consensus algorithms, but the BC is the only one that simultaneously enjoys the following three properties *(i) trustless*: There is no need to own a certified digital identity. The involved entities do not know each other, but they can anyhow exchange data without having to know their respective identities, *(ii) permissionless*: Nobody decides who can or can not operate on the BC network. There are neither permissions nor controllers and *(iii) censorship resistant*: BC being a network without controllers, where entities trust only the quality of the cryptographic algorithms that govern the operation, anyone can transact on the BC. A transaction, once sent and accepted, can not be stopped or censored. In addition, BC can be categorized into two types based on its functioning: permissionless and permissioned. A permissioned BC limits the actors who can participate in the consensus of the system state. In a permissioned BC, only a limited selection of users have the rights to validate the transactions. It may also restrict access to approved actors who can create smart contracts. On the other hand, permissionless BC allows anyone to join the network, participate in the process of block verification to reach a consensus and also create smart contracts.

After this critical distinction, it is essential to understand what BC attempts to solve. Let’s consider the traditional way to make economic transactions among individuals. Let’s assume that Bob wants to send money to Robert. To do this, a centralized Trusted Third Party (TTP) has to perform the following three steps: *(i)* Identify Robert’s state (e.g., Japan), *(ii)* Identify Robert’s bank account, and *(iii)* Move money from Bob to Robert. Exploiting the BC, it is possible to obtain the same result but with some improvement by *(i)* removing the TTP, *(ii)* making the transaction faster than three days: *immediate*, and *(iii)* making the transaction cheaper: Bitcoin transaction fees are voluntary.

BC technology is based on four central concepts: *(i) a peer-to-peer network*: this solution removes the central TTP implying all nodes within the network have the same privileges. In this network, nodes can interact with each other utilizing a pair of private/public keys. The private key is used to sign transactions, and the public key is used as an address to be reachable on the network. *(ii) open and distributed ledger*: Let’s imagine a ledger as an accounting book collecting all the transactions of the network in chronological order. This data structure is not a centralized entity, but each node has got its own copy of it. The ledger is open and public to everyone. Everyone on the network can see where the asset is and how much asset each one has in his/her account as well. Moreover, each node in the network can decide whether a transaction is valid or not valid. *(iii) ledger copies synchronization*: In this kind of scenario, where nodes have their own copy of the same ledger, a way to synchronize ledgers across nodes is needed. To accomplish such a goal, three main steps are required *(a)* to publicly broadcast the new transactions to the network, *(b)* to validate the new transactions, and *(c)* to add the validated transactions to the ledgers. *(iv) mining*: In a distributed system, there are network delays and not all of the nodes receive the transactions (block of transactions, to be precise) at the same time. Thus, there is a need to prevent every node from adding a transaction to the chain because the chain must only have a valid and ordered branch.

**Figure 1:**
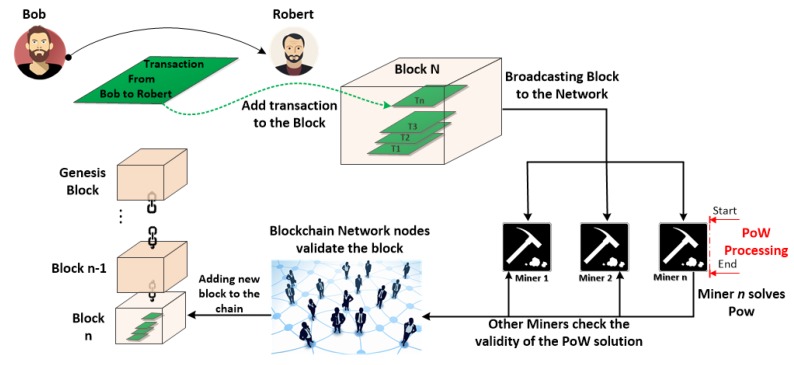
Transaction block validation and addition flow.

Miners are unique nodes that can add transactions to the chain. Miners are going to compete among themselves to understand who will be the first to take the new transaction, validate it and put it into ledger (chain). The first miner that will do that will get a financial reward. To be the first, a miner needs to validate the transaction and to solve a mathematical guessing game. In this way, only one miner at a time will be able to add transactions to the BC. Moreover, to avoid attacks to the system like the well known “double spending attack” [20], a solution to make the “game” hard for dishonest miners is needed. This solution is essentially to make it expensive (invest a lot of computer processing power) for adversaries to add transactions. This mathematical game is called Proof of Work (PoW) [21] and it is an operation of inverse hashing to determine a number (nonce) such that the SHA-256 hash of the pair “set of data,” representative of the block (set of transactions), and the chosen “nonce,” is less than a given threshold (see Figure 3). Figure 1 shows the steps needed to add a new block to the BC.

**Figure 2:**
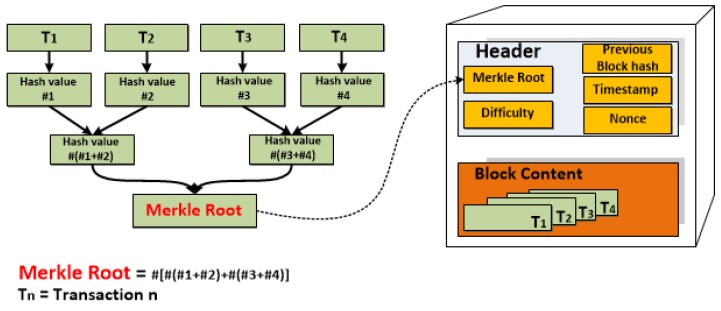
Block structure.

After realizing that a ledger is a chronologically ordered data structure of transactions, it is essential to know that it collects items called blocks. Each block is a set of transactions. More specifically, each block of the chain contains two elements (see Figure 2) *(i) Header*: It consists of a timestamp, difficulty target of the PoW, the hash value of the previous block HEADER, which is a cryptographic link that creates the chain and makes it tamper-proof, Merkle tree root, which encodes the transactions in the block in a single hash code with leaves signifying data blocks (in this case, transactions), and nonce, which is required for solving the PoW, at the same time preventing a replay attack. *(ii) Block Content*: It contains all the inputs and outputs of each transaction. The inputs contain the output of the previous transactions and a field containing the signature with the private key of the owner. This is the ownership proof of such an asset. The outputs contain the asset to be sent and the address of the recipient (the recipient’s public key). The recipient will be the sole user, able to spend that asset because only his private key can prove the asset ownership.

Thus, in simple words, every time a group of transactions is approved, which is connected to the previous block through a hash, a unique and immutable stamp that provides the guarantee that no one can tamper the recorded data. The only way to tamper the BC is to gain the 51% of the computing power of the whole network acting on the BC. Therefore, it is impossible for the individual to make changes to the ledger. This is a fundamental element: the decentralization of the BC is what makes it secure and distributed; moreover, such a decentralization allows the elimination of any central entity, relying instead on the “*democracy of computing power*,” which is assured by the thousands of participants in the BC.

### 3.1. Coins vs. Tokens

In general, all coins and tokens are considered as cryptocurrency even though there is a fundamental difference between coins and tokens. Most of the tokens are used in an altogether different fashion than as means of currency exchange. Cryptocurrencies are commonly characterized as *(i)* Alternative Cryptocurrency Coins (Altcoins or Native Tokens), and *(ii)* Non-Native Tokens. Altcoins or native tokens are coins that are built on their BC, usually similar to Bitcoin or Ethereum with changes to underlying protocols, thus conceiving an entirely new coin with a different set of features. Non-native tokens, on the other hand, usually reside on top of another BC and do not make any changes to underlying protocols.

Alex Kruger in his article classified digital assets into Native and Non-Native Tokens. Often discussed native tokens like Bitcoin, Monero, and Ether have their BC, while non-native tokens use other BC platforms. The author further classified non-native tokens into various classes: *(i) Protocol tokens*: The tokens which are based on protocols powered by rules of the BC, on which the tokens are built—for example, Augur [22]; *(ii) Utility tokens*: The tokens issued to utilize the services offered by any company. Usually, their tokens are offered as part of the ICO before the actual service goes online. ICO stands for “Initial Coin Offering” wherein a share of the cryptocurrency is offered to early investors in exchange for legal tender or other cryptocurrencies—for example, Factom [23] and Civic [24]; *(iii) Asset-backed tokens*: The tokens that are linked to a physical asset—for example, Tether [25]; *(iv) Participation tokens*: The tokens which are distributed among the participants to decide their share in future revenues without legal obligation—for example, DigixDAO [26], Crypto-One-Stop-Solution (COSS) [27], BitDice [28]; *(v) Partnership tokens*: The tokens which are distributed to decide the share of the shareholders. There may represent equity in the company—for example, Lykke [29]; *(vi) Appreciation tokens*: The tokens which are offered as appreciation—for example, Populous [30].

In contrast to cryptocurrencies like Bitcoin where the prices are determined by the supply and demand, a new class of tokens is emerging, called security tokens [31]. These tokens derive their value from an external, tradable asset and thus are regulated by federal securities. The potential applications may vary, with security tokens representing company stocks being the most promising. Compared to ICO, security tokens are issued based on regulation, which is easier and cheaper. Due to regulations, the legal risk associated with the tokens is reduced. The regulation also limits who can invest in them and how they can be traded. The typical features of security tokens [32] are as follows: *(i)* the markets will always be open irrespective of time or days (weekends or holidays), *(ii)* the interested parties can partner and acquire partial control over a high value asset, *(iii)* the swift resolution of settlement between trading parties as third-party like banks are not involved, *(iv)* there is a decrement in direct charge due to elimination of securities’ issuance costs and post-issuance administrative costs like ownership reconciliation, *(v)* programming terms of trade into smart contracts leads to automated compliance, and *(vi)* integration of standards like ERC-20 allows for accumulation of varying types of assets in the same wallet leading to asset interoperability.

### 3.2. Consensus

The consensus problem in BC, which is distributed and trustless, can be considered synonymous to Byzantine Generals Problem (BGP) [33]. The problem is formulated as how the generals can come to a common conclusion in the presence of a small number of traitors and miscommunications. Thus, the consensus protocol must exhibit the Byzantine Fault Tolerant (BFT) property. The BFT protocols are categorized as (i) Proof-of-*: The basis of these kinds of algorithms is the election of a leader. The leader is then responsible for validating the new block and propagating through the network. In case of conflict, resolution mechanisms are also present. *(ii) Byzantine Agreement*(BA) [34]: The basis of these kinds of algorithms is majority voting. All participants of the network are involved in validation, and when a threshold of nodes agree on a block, the block is added to the network. A major prerequisite is that the majority of nodes should be honest.

The *Proof-of-Work* (PoW) [21] is a computationally intensive mathematical problem that makes the BC what it is. In fact, it would be impossible to talk about BC without the existence of PoW. However, the PoW has been strongly criticized because it is considered excessively difficult, computationally heavy and very expensive in terms of energy consumption [35]. However, it is not possible to reduce the work linked to the PoW. The PoW must be tough (challenging) because, if you want to obtain a “value” (i.e., Bitcoin) as a result of a performed work, this cannot be easy to do. In fact, the native goal of the PoW is to avoid the spamming attack. Let’s imagine a Bitcoin network without PoW and a user that wants to generate a DoS. He could flood the system with new blocks thus clogging the network itself forcing all the nodes to perform extra work to find, among tons of spam blocks, the only one that is valid.

**Figure 3:**
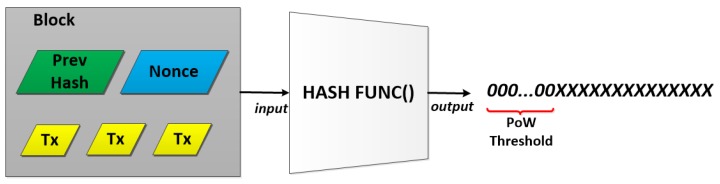
Proof-of-Work calculation and threshold.

Furthermore, the PoW must be an asymmetric task, meaning: hard to solve and very easy to verify. Specifically, a miner needs much time in finding the nonce that solves the hash puzzle, but the other miners in the network can immediately verify the validity of the found solution. Figure 3 shows an example of PoW. A Miner, to attach a block to the chain, must find a nonce such that the hashing of such a nonce and the data (block) gives a resulting value that is lesser than a specific threshold. This is usually a value starting with a specific number of consecutive 0. The quantity of consecutive 0 is determined by the difficulty to solve the puzzle, and it is dynamically adjusted by the network. Another important characteristic of the PoW is that anyone can do it. Any device, in theory, can solve the hash problem, and this makes the PoW a democratic system. Differently from the other consensus systems such as Proof of Space (PoSp) or Proof of Stake (PoS), where some nodes are cut off from the mining process because they do not have the basic needed requirements, with PoW any node can try to solve the problem. Moreover, PoW makes hard not only the addition of new blocks to the chain but also the alteration of previously added blocks. This means resilience and safety for the BC. That is why anyone who wants to cheat needs a much more substantial computational power than he would need if acting honestly.

The problem with PoW [19] is that multiple miners working towards a common objective lead to tremendous wastage of computing power and electricity. In addition, due to the requirement of high computing power, mining is advantageous if it is done in pools, thus defeating the decentralization. Unlike in PoW, where the fastest node is chosen as the leader, *Proof-of-Stake* (PoS) [36] elects a leader pseudo-randomly based on the “stake” presented by the participants. The malicious participant will lose his/her stake as well as participation in the future. In Casper [37], a node is required to place a safety guarantee, called bonding and thus becomes a bonded validator. After bonding, the validator can place a stake on which block will be included next. On acceptance, a reward will be received by the validator. Stakes against the consensus will cause the validator to lease his/her bet. Dishonesty will result in deletion of the bond.

*Delegated Proof-of-Stake* (DPoS) requires voting to reach to a consensus. The responsibility of network management is given to delegates who are not incentivized [38]. Their responsibilities include fee schedules, block intervals, and transaction sizes. Delegators can also propose changes which can be adopted based on the voting of the network. The responsibility of transaction validation and block creation rests with witnesses. Leader selection is deterministic and in a round-robin fashion. Witnesses are paid in terms of block generation and witnesses failing to do so are denied their privileges. *Proof of Authority* (PoA) is the successor of PoS, wherein the reputation of the validator acts as the stake [39]. The reason being that equal coin ownership does not essentially translate into equal motivation towards ensuring the honesty of the network as users differ in terms of their net worth. Reputation is difficult to regain once lost and thus is a better choice for “stake.” PoA networks have high throughput but are centrally-controlled by the validators.

*Proof of Capacity* (PoC) or *Proof of Space* (PoSpace) is an alternative to PoW, using miners’ hard drive space instead of computing power for solving cryptographic challenges [40,41]. Instead of working simultaneously to solve a problem in real time, miners perform the “plotting” operation wherein they try to store different solutions to different problems, thus eliminating the need for on-the-fly solving of the problem. The miner who can solve the puzzle fastest is elected as the leader for block addition to BC. PoC does better energy management compared to PoW. However, there is still a possibility that multiple users can collude to combine the storage power and thus centralize the network. Proof of Elapsed Time (PoET) exploits Intel’s SGX CPUs’ (Santa Clara, CA, United States) trusted execution environment for reaching to a consensus [42]. It was originally designed for a Hyperledger Sawtooth BC project (San Francisco, CA, United States), which is permissioned BC. PoET is a lottery-design consensus protocol in which SGX allows user-level code to act in a semaphore environment (enclave). Other waiting miners are provided a random wait time before allocating the semaphore region. PoET essentially works as follows: *(i)* every validator requests a wait time from an enclave (a trusted function); and *(ii)* the validator with the shortest wait time for a particular transaction block is elected as the leader. A major concern about PoET is that it is locked-in with Intel’s hardware. Researchers have also pointed out that SGX is not without security flaws.

*Algorand* is a BA class of algorithm [43]. It is similar to PoS in that account holders with larger shares have higher chances of becoming a leader. After each block addition, a one-time committee of block proposers is selected using a cryptographic algorithm for consensus. Here are the steps from committee selection to block generation *(i)* A random leader is selected, and he/she proposes and circulates a new block. *(ii)* A randomly-selected committee is chosen and reaches a BA on the block proposed by the leader. *(iii)* The agreed-upon block is digitally-signed by a certain number of committee members. *(iv)* The digital signatures and the identities of the signers, as well as the hash of the new block, are circulated so that everyone on the network can authenticate the new block. Compared with PoW, Algorand requires minimal computation power, and it does not fork with overwhelmingly high probability. A block that is entered into the BC is considered final. The Ripple network follows the *XRP Ledger Consensus Protocol* (XLCP) [44]. It consists of two kinds for nodes: *(i)* tracking nodes, and *(ii)* validator nodes. The tracking nodes are responsible for client transaction distribution in the network and querying the ledger. Some of the tracking nodes act as validators. Validators are responsible for validating and adding blocks to the BC. Here is how XLCP works *(i)* Client submits the transaction to tracking node. *(ii)* Tracking node relays the transaction to other nodes. *(iii)* Each tracking node is attached to set of validator nodes and decides based on the recommendation of its validators. *(iv)* When a supermajority (80%) of nodes approves this candidate transaction, this transaction will be included in the new “last validated ledger.”

*Stellar Consensus Protocol* (SCP) from Stellar is constructed as a Federated Byzantine Agreement (FBA) [45]. Instead of waiting for a threshold of total nodes of the network, a node forms a subset of trusted nodes on the network. The node takes a decision based on the consensus of its trusted circle. Thus, misbehaving nodes remain out of trusted circles and are eliminated from the decision-making process. In HyperLedger Fabric where *Practical Byzantine Fault Tolerance* (PBFT) is implemented, every transaction is validated by every node in the network [46]. However, the sequence of transactions in every node is not the same. A leader is thus elected to propose the sequence. All of the rest of the nodes then communicate with each other about the sequence until a consensus is reached. *Delegated Byzantine Fault Tolerant* (dBFT) leverages proxy voting [47]. In dBFT, nodes are elected to reach to a consensus on the next block to be added to the BC. These nodes are called bookkeepers. Voting, and therefore the selection of bookkeepers, happens continuously. Bookkeepers are required to register their identities with the network. Table 2 summarizes the comparison between various consensus algorithms. Transaction finality specifies whether the addition of transaction to the block is treated as final. With multiple blocks being mined simultaneously in algorithms like PoW and PoET, there is a possibility that mined blocks become part of a temporary fork and are thus rejected. The requirement of a cryptographic token is a must for algorithms like PoW and PoS as it is part of their design (incentive for work). Other consensus algorithms do not require them. Tokens serve another purpose of preventing spam or DDoS attacks. The trust model signifies if the identity of the participants is known. PoW, PoS and PoET models are based on cryptographic work and thus can be considered untrusted. Other algorithms assume trusted participants.

### 3.3. Blockchain Platforms

The vision of *Ethereum* is to create an unstoppable censorship-resistant self-sustaining decentralized world computer [48]. Ethereum is an ecosystem of nodes (computers) capable of replicating and processing data and programs called smart contracts on all nodes without a central authority. Ethereum can be thought of as a programmable BC. As against the Bitcoin transactions where the user operations are fixed, a user can create a complex operation using Ethereum. This extends the application of Ethereum beyond cryptocurrencies. Ethereum is characterized by Ethereum Virtual Machine (EVM) and smart contracts. Ethereum is “Turing complete” with the EVM at its core. The EVM is an isolated sandbox environment for smart contracts. The code running inside EVM is isolated from network access, other processes or the filesystem. The code of the smart contract has further limited access to other smart contracts.

*BigchainDB* is an open source system that is a combination of a big data distributed database and BC features like decentralization of control, immutable ledger, and management of ownership of digital assets [49]. The key features of BigchainDB are *(i)* Records are added to BC without Merkle Trees or sidechains. Sidechains allow digital assets to be securely transferred between two different BCs if the need arises. A sidechain is an alternate BC that is linked to another chain, usually called parent BC, using a two-way peg. The two-way peg mechanism allows interchangeability of assets between the parent BC and the sidechain. The original BC is usually referred to as the “main chain,” and all additional BC are referred to as “sidechains”. *(ii)* It supports transparency, custom assets, transactions, and permissions. *(iii)* It supports Federation Consensus Model. *(iv)* It supports both public and private networks. *(v)* It does not support any native currency. *(vi)* The permissions are set at transaction level. *Chain Core* is a BC platform dealing with financial assets on a permissioned BC infrastructure [50]. Chain Core is powered by open-source Chain Protocol. Control, transfer, and creation of assets are decentralized among the participants. A consensus among the participants is reached by a federation—a designated set of nodes. The key features include: *(i)* It supports native digital assets. *(ii)* It utilizes role-based permissions for operating, accessing, and participating in a network. *(iii)* It supports multi-signature accounts. *(iv)* It supports smart contracts. *(v)* It provides transaction privacy.

The *Corda* platform provides pluggable consensus, which is unique among all open-source distributed ledger platforms [51]. The key features include: *(i)* It does not require global broadcasting of data across the network. *(ii)* It supports pluggable consensus. *(iii)* It supports querying with SQL, join to external databases, bulk imports. *Credits* is a development framework which supports permissioned distributed ledgers [52]. It uses a variant of PoS. It is based on a leaderless two-phase commit algorithm with variable voting power. *Domus Tower Blockchain* is focused on regulated environments such as securities trading where participants know each other and can independently decide whom to trust [53]. Merkle DAG is the basis for data storage. A node capable of writing to BC has the authority to write transaction to that chain. The authorization model is centralized. The features include *(i)* The blocks are linked as assets of an account on one BC must match the liabilities on account of another BC. *(ii)* It is capable of a high rate of transactions in a scalable manner. *(iii)* There is a separate entry for tracking credit and debit. *Eris-db* belongs to a permissioned distributed ledger capable of executing Ethereum smart contracts on a permissioned virtual machine. The consensus mechanism followed is a Byzantine fault-tolerant Tendermint consensus engine, which is a deposit based PoS protocol.

*HydraChain* is an open-source system extending Ethereum for private and consortium chains in a permissioned way [54]. It uses BFT consensus protocol. The key features are *(i)* It is fully compatible with Ethereum protocol. *(ii)* The validators in the system are accountable. *(iii)* It does not support forks or reverts. *(iv)* It is possible to write smart contracts in Python. *Hyperledger Fabric* enables management of multiple networks capable of supporting different assets, agreements, and transactions between different sets of member nodes. Channels allow crypto assets to be derived from different certificate authorities. *Multichain* supports multi-asset financial transactions and is an open-source BC platform [55]. It is based on Bitcoin’s BC. The key features include *(i)* It natively supports multi-currency. *(ii)* It supports multiple networks simultaneously on a single server. The consensus mechanism is similar to PBFT with one validator per block, working in a round-robin type of fashion. *Openchain* manages digital assets and is based on an open source distributed ledger system [56]. The tokens are interoperable with Bitcoin with support for smart contract modules. It is based on partitioned consensus. *Lisk* supports the Sidechain consensus and was developed in Node.js [57].

### 3.4. Smart Contract

BC, being a decentralized system, eliminates the need for intermediaries, thus saving time and conflict. Nick Szabo [58] proposed utilization of decentralized ledger for self-executing contracts. These contracts could be converted, stored and replicated by participants of BC. Non-availability of necessary technologies, especially the distributed ledger, caused a hindrance in the realization of the concept at that time. After the appearance of Bitcoin [19] in 2008 and Ethereum [48] in 2014, it became possible to support the realization of a smart contract. A Smart Contract, in simple terms, is a digitized form of a legal contract. It consists of a set of protocols, which the participating entities should agree on, and conditions causing the executions of those protocols. The public availability of the code on the BC creates a trust in the participating entities, and automatic execution eliminates the need for a TTP. Smart contracts have the following properties *(i) Autonomy*: Participating entities consent on the decisions and thus need for intermediary and bias related to them is eliminated. *(ii) Trust*: Essential documents are present on a public ledger and thus cannot be destroyed or lost. *(iii) Backup*: Presence of data on multiple nodes participating in the network makes the data safe. *(iv) Savings*: Smart contracts eliminate the need for a TTP and thus save money.

### 3.5. UTXO

BC stores meta-data about transactions and user balances. There are two popular approaches on which two popular BC platforms, Bitcoin and Ethereum, are based on. The first approach is known as the Unspent Transaction Output (UTXO) Model and the second approach is known as the Account/Balance Model. Bitcoin is based on the UTXO model, while Ethereum follows the Account/Balance Model. Bitcoin and its associated transactions adhere to the UTXO model. The unspent transactions” are the group of transactions received by a user, but not yet sent by him. At any given instant, a user balance can be found by aggregating the outputs. BC will present these assets on different accounts owned by the user and tracked by wallet using user’s keys. The validity of a transaction is based on the proof of ownership. If a user can prove ownership, he/she can transact. Typical features of UTXO include *(i)* Higher degree of privacy: As the user uses a new address for each new transaction, it provides the user a pseudo-anonymity, thereby providing a higher degree of privacy. This feature is more relevant for currency, but, in the case of other digital assets, it is necessary to keep track of the assets. *(ii)* Potential scalability paradigms: UTXO compared to the account model is theoretically compatible with certain kinds of scalability paradigms. The owner forgetting data harms only himself and do not affect the network in contrast to the account model, where everyone losing the portion of a Merkle tree corresponding to an account would make it impossible to process messages that affect that account at all in any way, including sending to it. However, non-UTXO-dependent scalability paradigms do exist.

## 4. Use of Blockchain in Smart Environments

In our opinion, BC represents the missing piece of a puzzle to solve privacy and reliability flaws in IoT. The intrinsic decentralized, autonomous, and trustless features of the BC make it suitable to be applied in several different scenarios such as “Smart Home,” “Smart Industries,” “Smart Grid,” and “Smart City” as well. For example, the BC could keep an immutable history of smart devices. Moreover, it may enable an autonomous functioning of intelligent devices, removing the presence of centralized authority or human control by the use of smart contracts. Furthermore, BC can also create a secure way for smart devices exchanging messages with each other. Therefore, the goal of this paper is to figure out how BC can meet the IoT security and privacy requirements or in general how BC can be integrated with IoT.

**Figure 4:**
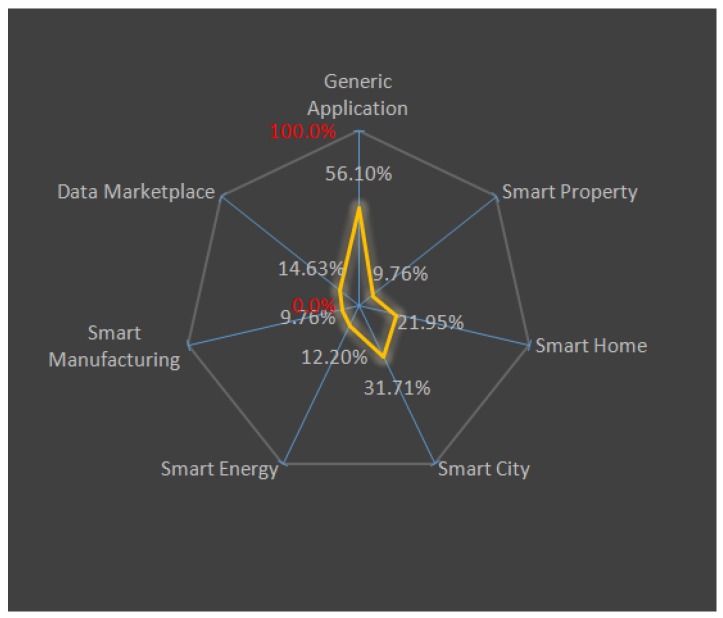
Paper scope distribution.

Thus, in this section, we are going to categorize the analyzed papers in four main groups, taking into account the field where each of them operates. The considered groups are Smart City, Smart Home, Smart Property and Generic Context. The Smart City is further categorized into two sub-sets: Smart Industry and Smart Grid. In Figure 4, a graphic distribution of the papers under each of the subsets is presented. Table 3 represents this categorization. Table 4 categorizes the surveyed solution into two categories *(i)* data manipulation, and *(ii)* device manipulation. A data manipulation approach utilizes the BC as a secure repository exploiting its features like an immutable public ledger and ability to create a digital trail for verification. A device manipulation approach, on the other hand, utilizes BC not just as a secure book of records, but utilizes smart contracts to create autonomous systems capable of making decisions on the basis of business logic. Smart contracts also eliminate the need for a trusted third-party as the rules are executed automatically based on present conditions and rules are publicly available thereby promoting transparency. Another organization of the analyzed frameworks has been done in Table 5. In this table, the frameworks have been split into three subsets considering their development level. Table 6 presents a categorical view of solution addressing the challenges presented in Section 2. Table 7 tries to give another perspective to the survey focusing on specific use-case and goal specifying the “technologies” or “models” used instead of considering the operating environment of the solution. Table 8 specifies the consensus algorithm adopted in each of the presented solutions.

### 4.1. Smart City

A city can be understood as a Smart City if it is capable of intelligently managing economic aspects, mobility, citizen relations, environmental resources, etc. From the infrastructural point of view, a Smart City is designed to provide services to citizens and enterprises through communication and information technologies. From the technological point of view, this typically means having a network of sensors or in general “smart devices” that can capture data from the surrounding environment and make it available to citizens and authorities for optimal and real-time management of the city. It takes place through the interconnection of infrastructures and devices such as smart energy meters, safety devices, home appliances and smart cars or video surveillance systems. It is easily conceivable that the more “intelligent” and interconnected a city is, the more it becomes a desirable target for hackers. A Smart City management is based on a continuous data exchange among smart devices which collect such data from citizens and the environment.

The risk of an attack on critical services of a smart city is very high and, if it occurs, could lead to serious damage to the citizens’ privacy. In fact, the current scenario confirms that cybercrime attacks are a certain element of the ICT world: insofar as ICT pervasiveness grows, so does cybercrime, and that means that the latter is steadily on the rise. To tackle these risks, adequate defense and protection systems, dealing with any critical attack, are needed. Some security measures useful for reducing cybernetic risk are: encryption, anonymization, and pseudonymization of data or application of the “*security by design*” [117] approach.

An expedient for separation of collected data (that are therefore also physically located in different servers) is the pseudonymization, so that only through a joint treatment of the data is it possible to get the identification of the producer; pseudonymization thus does not preclude that, by the merging data from different sources, the subject becomes identifiable again. In other words, personal data may no longer be attributed to a specific individual without the use of additional information. This additional information is stored separately and, to ensure that such personal data is not attributed to an individual’s identity, it is properly and technically organized. This exactly is the goal of Biswa et al. and Conoscenti et al. [63,66]: to find a possible approach to overcome the data privacy risks from the different point of views.

The papers presented in this subsection propose several BC-based systems that operate in such a context. The first is based on pseudonymization concept. In fact, their solution is to split data into several chunks and to distribute them among several smart devices in an IoT environment (Smart Home). In this way, only the owner can rebuild the original data. Moreover, the use of the BC technology provides certification of the data. The BC contains the hash of the data produced by the IoT devices. An owner of smart devices can specify access rules to the data.

Thus, if some external entity (Service Provider) wants to get the data, it has to be authenticated. The data owner decides, using a specific access list, whose public key is allowed to access the produced data [63], instead proposes the integration of BC into the different layers of the Smart City framework, namely, physical, communication, database, and application layers. The proposed framework overcomes the limitation present in each layer via the BC technology. Ethereum is responsible for providing smart contract functionalities with BC as distributed DB. Finally, the application layer could integrate security to avoid granting intruders any access to other dependent processes.

Another solution that exploits the Telehash protocol for the communication is that developed by Filament company [59]. The Filament idea is to create wireless networks to control any system, from the lights of a city to the alarm of a company. The system is based on the BC and smart contracts’ technologies, and it enables smart devices (sensors, smart appliances, etc.) to discover, exchange messages, and interact with each other autonomously and without any central entity. Before any communication, the devices must authenticate with each other, e.g., by the Secure Socket Layer (SSL) or Transport Layer Security (TLS) protocols, possibly based on public key infrastructure (PKI). In addition, Prabhu et al. in [84] proposed an intercommunication model between smart devices in an IoT environment that makes use of the BC as a backbone. The idea is novel compared to those proposed in [59,63]. The IP address acts as a key to retrieve information stored on BC. In addition, events stored on BC are used as notifications.

Additionally, on the topic of secure communication between IoT devices, the startup Moeco (Berlin, Germany) proposed a platform called Moeco [93], named after the company. In an IoT environment where many peers are involved, and some of them play the role of hops, private communication between two nodes must be realized. In light of the above, the Moeco platform is exploiting the BC to develop its own IoT data routing platform thus aiming to create a new concept: Domain Name System (DNS) of things. Every node (e.g., mobile devices) can install Moeco software, becoming a gateway node (bridge) of networks and is incentivized for fulfilling tasks. The payment is in a token called MoeCoin (MOE). The BC used is Ethereum-based, and it stores all the connections and data transfers within transactions. The payment is signed and processed on the BC as well. However, Moeco is planning to move towards a different consensus algorithm, namely the Exonum [116] custom-built Byzantine one.

Similarly to Biswas et al. [63], Hashemi et al. [72] proposed a multi-layer BC-based framework. The target is the same as the previously analyzed works: Data Protection. However, the authors followed a publish–subscribe approach to create a secure environment. The authors assert that conventional best practices to grant data security are not suitable in IoT: the access control list (ACL) cannot be placed on sensors, Kerberos is a central point of trust, etc. The presented solution is based on two concepts: separate data store from data management and design components in a scalable, decentralized and distributed way. The three layers are: *(i)* a data storage system based on BC to provide persistent distribution and transparency; a *(ii)* messaging service providing a scalable communication system between senders and receivers; *(iii)* data management providing a mean for the interaction between roles (data owner, data source, data requester, endorser) with an access control mechanism. The BC is used to collect access control data in a decentralized way. The system allows users to access the data in three different manners: *(i) direct access*: everybody can access the BC and download all the chain. It is easy to implement but not feasible in all the contexts because it requires much computational power in each node to manage that large amount of data. *(ii) Client-Server* solution is feasible if there is enough trust between clients and server. *(iii) pub-sub method* represents a solution where the “Publisher” does not have access to the data. It will send an encrypted version of the data to the “Subscriber” who will be able to decrypt it.

A challenge that researchers are trying to resolve is to find a suitable way to implement access control and authentication approaches fulfilling the IoT requirements. We just saw that classical ACL protocols do not fit the requirements of IoT environment and this is true for other approaches such as Mandatory Access Control (MAC), Discretionary Access Control (DAC), and Attribute-Based Access Control (ABAC) because of their centralized nature. Deters in [92] proposed a novel model to achieve access control in IoT. The model evaluates the suitability of BC based on statistics derived from access patterns. The first is the “Announcement” approach in which the owner of data or resource can send the transaction to the BC containing the address and his privilege. The second approach is based on a Smart Contract. To access the data, a user must send a transaction to a given smart contract, which, after evaluating the access control rules, decides whether to grant or deny permission to the user.

Even though the purpose of Aitzhan et al. in [60] is to solve the privacy and anonymization problems by means of BC, what is novel in this paper is the scenario. The target is not hiding the identity of “data” producers but of energy sellers. Thus, in this case, the paper is placed in a smart context that may be considered a subset of the Smart City, namely Smart Grid. The BC is integrated into PritWatt to provide privacy and security to the energy trading system. In such a context, a transaction is understood as an exchange of ownership tokens. The proposed energy trade system generates and uses new addresses for each new attempt to sell energy. In this way, the user’s identity is hidden every time it generates a transaction. The trading process consists of three actors: *(i) a prosumer (PROducer and conSUMER)*; *(ii) a consumer*; and *(iii)* an energy broker called *Distribution System Operator* (DSO). The DSO manages security and avoids double spending of the energy. Thus, this work is categorized under the smart grid label in Table 3, in turn, a subset of the smart city category. Moreover, it is possible to insert this work into the smart property context. In fact, the system considers the exchange of tokens about the ownership of a specific amount of energy. The authors also proposed a proof of concept of the presented system.

The secure automation of the energy exchange is possible if smart contracts are exploited. In fact, it is possible to trigger an energy transaction if specific conditions between prosumers and consumers are satisfied. This is the focus of Munsing et al. [79] where smart contracts are exploited to optimize the energy distribution within micro-grids. In addition, Lombardi et al. [78] worked on the same topic but from a higher level point of view. Specifically, they presented a three-layer BC-based system that exploits smart contracts, policies and auctions in a grid. The system improves security, availability, and reliability reducing the transaction costs. Together with the previous works, Nehaï et al. [80] also aim to integrate SmartGrid and BC. The authors utilized BC for peer-to-peer exchange of electricity, at the same time optimizing its transport. In particular, the idea is to move from a centralized approach to a peer-to-peer solution. To make BC work well in a SmartGrid context, they presented “ElectricChain” [113], which is a mix of different BC approaches. The main idea is to manage transactions related to the sale of electric energy between two users belonging to the same micro-grid. Inhabitants can exchange energy produced in their homes with others, earning a specific amount of “coins” (SolarCoins [118]) per kWh. The system is based on the exploitation of smart contracts containing all the rules and agreements by the inhabitants. Moreover, for efficient transaction management, a smart contract owns the consumption patterns of the BC users. The authors further highlight the flaws of the presented approach. In the following, the focus is slightly moved to another application field that, in our opinion, is directly linked to the Smart City. It is called Smart Manufacturing. The Smart Manufacturing/Industry can be seen as the adoption of digital technologies capable of improving the interconnection and cooperation of resources used during the operational processes distributed along the value chain [119]. Other more comprehensive definitions about Smart Manufacturing are presented in [120].

It is in such a context that Bahga et al. [62] gave a novel Cloud-based Manufacturing (CBM) system, called BPIIoT, exploiting the BC technology to make it decentralized and trust-less. BPIIoT can improve the well known CBM (which is a new manufacturing paradigm that aims to provide manufacturing resources and capabilities as a Service) platforms by exploiting BC and smart contracts. Each IoT device is a node of the peer-to-peer network and has an account on the BC. A user of the system can transact with the machines directly enjoying on-demand manufacturing services by sending transactions to a registered machine. BPIIoT can be exploited for several manufacturing applications, such as *(i) On-Demand Manufacturing*: users transact directly with manufacturing machines such as (Computer Numerical Control) CNC machining or 3D printing; *(ii) Smart Diagnostics & Machine Maintenance*: Exploiting the sensors’ values, it is possible to check if a specific machine is working fine or not; *(iii) Supply Chain Tracking*: by tracking product ownership through different phases in a supply chain; *(iv)* and others. Moreover, the authors proposed a use case: machine maintenance and smart diagnostics application. The system is based on Ethereum, and the contracts are developed in Solidity language. The benefits of this choice are embedded in the BC technology, and, consequently, the system suffers from the same problems as Ethereum, namely: smart contract vulnerabilities, privacy, efficiency, and government regulation. In fact, in the paper, the authors did not face problems with user anonymization or devices’ provenance. This is, instead, the subject of [71]. The authors aim to grant and certify the provenance of a constrained device without revealing its identity.

The proposed architecture is based on several security concepts such as Enhanced Privacy ID (EPID) that in turn is an extension of the Direct Anonymous Attestation (DAA). The five actors of the system are *(i)* device manufacturer, *(ii)* constrained device, *(iii)* device owner, *(iv)* IoT Data Broker (provenance verifier) and *(v)* a BC p2p network. The device manufacturer sends to the IoT Data Broker a public key to check the provenance of a group of devices. Each device has the corresponding provenance issuing private key. Each device calculates another pair of keys to sign the BC transactions. In this manner, the manufacturer cannot monitor the device activity because it does not know this new pair of keys. Another analysis about the BC roles in supply chain management is given in Kshetri et al. [121]. The authors explain how BC can be merged with the IoT and the aspects of the supply chain it can improve. Remaining in the Smart Industry context, there is an aspect that was not included in the papers cited previously, namely: the smart energy exchange. The scenario presented in Sikorski et al. [86] considers a novel machine-to-machine (M2M) communication model based on the BC technology. The machines or components, in the Industry 4.0 [122], must be able to talk with each other and trade every type of commodity (steam, natural gas, coal, energy). Every machine has an embedded system having its digital identity, and this allows for transacting over the BC. The presented system consists of three primary entities: the BC; an energy producer that, utilizing a transaction to the BC, publishes an energy offer; an energy consumer that picks up the best-published offer and then sends to the BC a transaction payment for that offer.

### 4.2. Smart Home

A smart home is, by definition, a home able to leverage an integrated home automation system, to enhance the comfort, safety, and consumption of people who live there [123]. The smart home enables owners to manage many internal functions even from outside the home. It is possible to program, activate, deactivate, and control the devices within it without necessarily being physically at home. Thus, a smart home provides the residents with the ability to optimize energy loads, create custom scenarios, and adapt the home to the owner’s preferences and habits. Through the previous Section 4.1, we analyzed different solutions aiming to reduce privacy and general security issues in a Smart City. However, what happens if the security problems in IoT occur in the home? Does the end consumer have the required knowledge and tools to defend itself from possible external attacks? There are several possible kinds of attacks that could be attempted by an intruder to obtain access to confidential and private data. To cite some of them: malware acting as backdoor; Man-In-The-Middle in case of unencrypted communication protocols; and merely obtaining access to the home router devices (hacking the password of the device). In many cases, breaking into a single device gives the hacker the possibility of violating others. Moreover, some studies demonstrate that, even when the sensor generated data in a Smart home is encrypted, it can reveal a great deal of information about the activities of the users. This is possible by just analyzing their meta-data and traffic patterns [124]. In light of the above in this subsection, we grouped all papers proposing solutions to solve some of the previous threats through the exploitation of the BC.

In fact, if we think about scenarios where users use private/public keys to sign and secure their activities or action in an IoT environment, even if data or the communication is ciphered, it is still possible to obtain information about the involved users or entities. This is because the public key of the user is known to anyone. Furthermore, there are several situations where the fact that two entities are talking to each other can be sensitive information. A solution to this problem is proposed in [61]. The main idea is to apply a Blockchain-based PKI system to those scenarios in which a PKI is needed. The application of this kind of solution can create a privacy-aware PKI, overcoming all the limits concerning the conventional PKI systems. A possible scenario in which to exploit the Blockchain-based PKI is the IoT. Here, a single user may act on multiple devices (smart TV, fridge, for example) and the linking of identity, being used across devices, could be a privacy concern. However, this paper does not explicitly focus on this scenario, but it provides an in-depth background about the BC architecture and Blockchain-based public key infrastructure (PKI) system. Another generic and similar solution to solve authentication issues is presented in Fromknecht et al. [69,110]. This solution is called CertCoin. It is a NameCoin based system that stores domain information together with their associated public keys in a public ledger (BC). NameCoin is an implementation of the Bitcoin protocol to create a completely peer-to-peer DNS system. Through the Namecoin system, the translation of domain names into the corresponding IP addresses takes place without the use of central servers that can theoretically be subject to government censorship. This solution puts together the pros of both Transparent Certificate Authorities [125] and the Web of Trust [126]. The proposed solution does not focus specifically on the application of CertCoin in IoT environment, but is a general approach. A similar approach is proposed by Axon et al. [61].

However, in our opinion, it can be exploited to manage security aspects in an environment composed of thousands of devices that must trust each other. Another “more efficient” solution than PKI or Pretty Good Privacy (PGP) WoT [126] is proposed in [77]. This solution is called Authcoin, and it is based on four main steps: *(i)* key generation, *(ii)* user–key association, *(iii)* public key formal validation and *(iv)* domain, certificate, and e-mail validation and authentication processes. The paper takes PGP WoT as a reference point, but Authcoin can be applied to any other authentication system. During the first step (key generation), a new key pair for the user is generated. Specifically, a user uses a local client that must be PGP-compatible. He can also add further information to the key-pair like email address and domain names. The second step merely checks if the public key meets specific requirements. If the requirements are met, the *(v)* validation, and authentication steps start. This is the most crucial step of the Authcoin flow. Its strong point is the “bidirectional request–response” challenge. The solution of the challenge is the proof of the responder identity. The authentication idea is based on the concept that a user cannot lie about his/her own identity because this is publicly available in the BC and everybody can easily verify it.

Moving from the specific authentication concerns to other kinds of attacks, it is interesting to cite Huh et al. [74] who propose a solution to make a denial of service and forgery attack-resistant smart home. The solution adopted is similar to Axon et al. [61], which is to manage and control devices by using the key stored securely within the BC. However, in this paper, the crux is the smart contract. The main idea is to be able to automatically change the device working mode, switching to energy saving mode when the energy consumption exceeds a specific threshold. This is obtained by exploiting smart contracts registered on the BC. To simulate an IoT system, they considered only a few smart devices, namely three Raspberry Pis and one smartphone. The Raspberry boards were used to meter electricity usage of home devices while the smartphone was used to configure the policies into the BC. This is possible using Ethereum smart contracts. There are mainly three simultaneous running processes on the BC: *(i)* homeowner sets up or changes working policies on the BC (sending data to BC); *(ii)* devices read the BC periodically to retrieve the updated policies; finally, *(iii)* the devices send electricity usage data to the Ethereum BC. To achieve such a goal, the authors wrote three smart contracts to manage the three processes described above. The authors found some weakness during the testing phase regarding the latency due to the Ethereum transaction validation process and the lack of possibility to implement a light client on Ethereum. The latter leads to a significant problem: how and where does the BC have to be stored?

As previously mentioned, smart homes collect and analyze a lot of potentially useful data. Just thinking about the number of connected devices continuously generating data may reveal users’ behavior. This digital information is critical knowledge that could be maliciously used by hackers. Therefore, it is easy to understand that the level of risk associated with a possible privacy violation is proportional to the number of smart and connected appliances. Moreover, there is a situation where only the fact that multiple smart appliances are running, therefore sending data, could be translated into the user presence in the home. If some malicious person has access to this information, he could physically break into the house during the absence of its owner. Wu et al. in [102] propose an out-of-band two-factor authentication scheme for IoT devices based on BC infrastructure. The proposed method uses Eris BC as the basis. The method utilizes the ability of secondary authentication factor to distinguish a home IoT device from an intruder even in the case when the access token is intercepted. The core idea of the out-of-band secondary authentication is to verify whether an access requester locates within the home or not. The secondary authentication is based on an out-of-band channel like the amount of ambient light in the home. The outside adversary has no control over the indoor lighting conditions. Hence, the verifier device will not get the right action code, and the adversary will fail the secondary authentication. The verification result will be recorded on the specific address on BC.

Dorri et al. [67,68] designed a multi-tier architecture to provide security and privacy to an IoT environment accordingly with the IoT requirements and overcoming the limits of the BC and those of the conventional security and privacy approaches as well. They applied BC, whilst removing the PoW, which is computationally intensive, and the coin concepts, but granting at the same time confidentially, availability, and integrity of the data. They used a system based on three layers: smart home, an overlay network, and cloud storage. The Smart Home is composed of devices and only one miner. The miner manages the BC and the access policies on the data. When a device is added to a smart home, the miner creates a block of that node and registers it to the BC. The block contains two headers, namely a block header and policy header. The first includes the link to the previous block in the BC; the second provides information about who can access that data. Each device can securely communicate with another through a shared key. The miner manages the distribution and creation of these keys (Diffie–Hellman algorithm). A device can choose to store data into local storage employing a shared key, or it could place data in cloud storage. To do that, a device has to send a request to the local miner that in turn will send a transaction to the public BC signed with the device’s key and addresses to the cloud storage. The presented architecture can meet five security requirements of a smart home: *(i) confidentiality*: by means of the use of the symmetric key encryption; *(ii) integrity*: using hashing algorithm; *(iii) availability*: just reducing the allowed transactions; *(iv) user control*: by the BC technology and finally *(v) authorization*: exploiting shared key and authorization policies.

Nevertheless, BC is not used with the unique goal to make a vulnerable environment secure. In fact, using smart contracts, it is possible to find a novel solution to existing problems. For example, a novel implementation and incorporation of BC into IoT is represented by Slock.it [13]. It represents an automatic authorization approach to access smart devices “*on demand and financially incentivized*.” This solution, based on Ethereum framework, aims to give users the possibility to control smart and real-world physical goods (called Slocks) employing the BC. The main idea is to make it possible to rent the use of compliant smart devices (an intelligent object with embedded Slock.it technology) in a smart home environment to external individuals in a trustless and automatic way. This usage (shared or rented) is regulated through smart contracts that accept payments, for that usage, without any intermediaries. A smart device owner, aiming to share or rent it, has to create a smart contract setting the price for the rental. One who wants to access that object first needs to find the contract and then needs to send a transaction payment on Ethereum to use it. The payment will trigger the smart contract that thus allows the access to the object (unlock the object). The end user will have back in its Ethereum wallet the difference between the rental price and the deposit he sent to the smart contract.

Wilkinson et al. propose another similar idea [87]. The project, called StorJ, exploits the BC technology and a p2p protocol to provide secure, private and encrypted cloud storage allowing users to rent the unused hard disk space of their computer. In the beginning, the system was based on the Bitcoin network. However, later it evolved, moving towards the Ethereum platform, exploiting a novel token called Storj Coin (SCJX). Users can rent their private free space storage (personal computer) belonging to other clients in the network and paying for that using SCJX.

### 4.3. Smart Property

The most famous BC system is Bitcoin [19]. This system enabled functionality never previously available in computer science, and the digital currency is just a first application of that technology. The Bitcoin network empowers the ownership and the anonymous transfer of digital coins, Bitcoins. However, Bitcoin software architecture allows for associating to an address a small amount of data (meta-data) that can be used to describe an “asset” different from a Bitcoin and the instructions to transfer such an asset from an address to another. In other words, this meta-data defines a new kind of digital coin that can be thought of as a “token.” These digital tokens are defined as “colored coins” (cc) and are associated with a value that corresponds to an object or service in the real world. You could, for example, have a cc representing your house or your car. Consequently, if/when you want to sell your house/car to another individual, you should send the cc to the new owner. In this way, there would be no need for a physical deed because the proof of ownership is in the BC. This solution to digitally manage the ownership of real goods takes the name of “smart properties.”

The smart property is strictly linked to the smart contract. In fact, in the beginning, the smart contract had to manage the simple activation or deactivation of a software license according to some straightforward conditions. The software license was in fact managed by a digital key allowing the software to work if the customer had paid the license. What BC did, and is doing at the moment, is to allow having guarantees regarding trust, reliability, and security that in the past were necessarily delegated to a “third” party. This is what Herbert et al. tackled in [73]. They presented a BC base system to achieve the software license validation by means of a peer-2-peer distributed network. The main goal of such a system is the improvement of the protection level provided by the standard software copyright. This work presents two interesting license validation models: *(i) Master Bitcoin Model* and *(ii) Bespoke Model*. Both of the models are a specific use of the smart property concept. The first one uses a couple of values address/Bitcoin as license ownership. The software is registered in the BC. The vendor charges this software account with a number of Bitcoins that when sent will represent the ownership of that software. The second one is based on the concept of the token. The token is a digital signature representing the entitlement to use specific software. The user that owns this token is allowed to use it. The authors underline the possibility to use this approach in IoT contexts. In this context, where the devices should be able to self-manage, mechanisms to auto-update and auto-validate software license are needed. However, these are static solutions that do not leverage smart contracts. The basic premise of a fully decentralized system is failing in this case. In fact, the authors talked about the possibility of adding further dynamic capabilities by means of smart contracts. A similar approach is followed by Ghuli et al. [70,90]. In the first, a method to decide ownership of IoT devices in a p2p manner is proposed. The described solution consists of transferring the ownership from one user (typically the device’s manufacturer) to another one (typically the end user) making a signed transaction (payment) to the manufacturer to obtain the ownership of the physical device (another transaction). These two transactions are added into the BC and verified by the other peers. After that, the manufacturer will send the physical good to the buyer. However, in the presented scenario, there is a central entity called a Certificate Issuing Authority (CIA) that issues a certificate (set of public key and private key) to any requesting entity and provides verification services of a digital signature using the signing authority’s public key.

Differently from the aforementioned two papers, Zhang et al. [90] in their work allow an exchange of two kinds of goods: real objects and digital ones (data). Moreover, they added flexibility and automation capabilities by means of smart contracts. Specifically, the authors presented a novel Blockchain-based architecture able to manage the transactions in the IoT. In particular, the authors focus on two types of commodities: *(i)* paid data (sensors’ acquisitions); *(ii)* smart properties (car, house, etc). To accomplish such a goal, they consider a new kind of cc to represent physical goods. For example, to describe the object car, you can use car-coin. The system counts two types of transactions: data and properties. Regarding the data, a user can pay Bitcoins to buy IoTCoin and then use IoTCoin to obtain data or asset ownership. The IoTCoin is a special cc: crypto coins that are based on the BC and can represent virtual goods. Regarding the smart properties, there is an exchange between object-coins and other object-coins or with Bitcoins. To access the IoT data, there are two ways: Positive and Negative. In the positive approach, with a p2p connection between user and provider, the user obtains IoT coins and a key for assessing the API offered by the provider in order to get the data. In the negative approach, the user sends Bitcoins to the data provider and receives IoT coins and the encrypted data. Another interesting analyzed use case is the car’s ownership exchange. The car’s engine is locked, and it can be unlocked only by the private key of the owner. This because, at the end of the transaction, the car control unit will contain the public key of the owner with which the car-coin (IoTCoin) is associated.

Table 5 refers to the development level of the solutions presented in the paper. We categorize a particular solution in a given development level. Many of the surveyed solutions such as BPIIoT [62], Cha et al. [65], Dorri et al. [67,68], CertCoin [69,110], ChainAnchor [71], Hashemi et al. [72] and Sapphire [89], are at a prototype level as they are open to users for evaluation and feedback, but are not yet at commercial level. Solutions like PriWatt [60], IBM Adept [64], Huh et al. [74] and Sikorski et al. [86] are at a theoretical level and do not present a Proof-of-Concept. Projects like IBM Hyperledger [75,76], IOTA (TANGLE) [82,83] and Storj [87] are at pre-product level as the product is available, but active research is still pursued. Products like TransActive Grid [10], Filament [59], Enigma [91,111] and Moeco [93] are more mature and thus are categorized as products.

## 5. General Solutions

In this section, works proposing generic solutions are grouped. These solutions can be applied to each field of the IoT environment. For example, Wörner et al. [88] propose a system allowing sensors to exchange Bitcoins with data. Each node has an address which is the Bitcoin’s pub-key. Essentially, when a client requests data from a sensor after discovering it within a sensor repository, he/she sends a transaction addressed to the public key of that sensor (including Bitcoins). The sensor will respond by sending a transaction to the client including data. This approach, in fact, is a generalization of the solution presented in [90] and thus it is only a possible application of such a technology to enable purchasing of sensor data in IoT. Taking into consideration what was discussed in Section 4.1 about pseudonymisation techniques, now it is interesting to introduce the Enigma framework. It exploits a similar idea entirely: to distribute data among several nodes and separate data from its references. Moreover, besides making it hard to rebuild the original version of data, Enigma adds an additional level of security: encryption of such data chunks (Zyskind et al. [91]). Thus, Enigma is a peer-to-peer network allowing different parties to store and operate on the data at the same time keeping that data completely private. The main components are: *(i)* a data storage using a modified version of a data structure called a Distributed Hash Table (DHT) for storing shared secret data chunks; and *(ii)* an external BC to manage network monitoring, access control and identities.

Thus, BC acts as a log of attempts to tamper the events. The DHT contains references to the data but not the actual data. Each node stores only a part of the data. Calculation on the data is spread over the nodes, and it is done without decrypting the data. Thus, the Enigma’s primary goal is to protect the data-in-use, but it also considers access control management and processing on encrypted data by means of the homomorphic encryption. This is a general purpose system that could be adapted to collect, manage and use data coming from IoT devices in a decentralized and trustless fashion. However, the exploitation of the homomorphic encryption and data computation within IoT devices is currently a tough challenge, and it must be deeply investigated. In this case, the BC has only a “surrounding” role in the presented scenario. It only plays a monitoring role and does not keep any data chunk or other information about it. The same approach to the use of BC is followed in Shafagh et al. [85] where the authors propose a BC-based storage system for IoT data. They implemented a layered storage system with the purpose of giving the end users control over their data and to ensure data ownership securely. The designed system consists of three layers, namely: *(i) data plane* based on the cloud and in particular on off-chain Decentralized Hash Table technology. This layer collects IoT data exploiting the DHT advantages in terms of scalability, bandwidth and storage requirement. In contrast to Enigma, DHT, in this case, stores key-value pairs. The value is the data chunk, and the key is the resulting hash value of the following four values: stream-ID, device-ID, user-ID, #counter. *(ii) The control plane* is based on the BC technology. The BC manages the access control to the data stored in the DHT. Each transaction, added to the distributed ledger, contains information about the data owner, data readers, corresponding data stream, and some additional metadata. When a DHT node receives a data access request, it interacts with BC to check if the requester has the permission to retrieve such data. *(iii) IoT devices and services*.

Another novel system aiming to perform analytical tasks on data is presented by Xu et al. [89]. The proposed system is called Sapphire and it utilizes the BC as a storage system. The main idea is to exploit the IoT device computing power to execute software (smart contracts) to perform computation on the data coming from a smart environment (cities, grids, and buildings). In this way, the benefit is twofold: (i) to reduce the data transfer on the IoT network, and (ii) to improve the execution time by utilizing parallel computation over a distributed storage system. Similar to IBM’s ADEPT [64], Sapphire consists of three different types of nodes, namely: super, regular, and light nodes. This classification is made to assign to each kind of node different tasks according to the resource available to them. For example, the light nodes have low resources and thus neither store all the BC nor perform analytical tasks on IoT data. The IoT devices (super and regular node) run algorithms on data and send the final result back to the users. Ouaddah et al. [81] also proposes such a type of BC usage. The authors here utilize the concepts of “Token” and “Smart Contract” to achieve their goal. The proposed access control model is called FairAccess and uses a “smart contract” to enforce access policies and to inform authorization decisions. One of the important concepts in BC is the UTXO.

It is a value locked to a specific owner. The UTXO consumed in a transaction is called input and the UTXO created by a transaction is called output. The authors’ idea is to create an authorization token that is a digital signature giving the access right to a recipient of that transaction. The flow works as follows: A user “A” sends a request to a resource owner (RO) “B.” The RO first creates a token encrypted with the requester PUBk and then defines access policies by utilizing the RO-wallet that works as PEP. PEP transforms policies in an “unlocking script” into GrantAccess Transaction and broadcasts the transaction on the BC network that works as a Policy Decision Point (PDP). In the end, the transaction is added to the BC, and the token is recorded in the requester-wallet. To obtain the access to the resources, the user “A” initially scans his wallet to get the token and creates a transaction “GetAccess” afterwards to obtain the access to the resource of device “B.” In the input of this transaction, “A” inserts the UTXO = Token of a previous GrantAccess transaction recorded in BC. When “A” tries to access resources of device “B,” the latter can verify the validity of the token. In addition, Cha et al. [65] designed a system to preserve the user’s data privacy by means of an Ethereum-based BC platform. Such a system avoids the situation where a user’s personal data could be obtained without the user accepting the privacy policies of an IoT device (specifically Bluetooth Low Energy (BLE) modules). The authors propose two examples, namely: wearable devices (intelligent glasses, Heartbeat sensors) and smart factory devices (production line machines). However, the presented solution can be applied to almost every IoT based context. The main idea is the introduction of a BC gateway entity that interacts with the IoT devices on behalf of the user. The interaction is based on smart contracts. The BC stores two types of smart contracts: one for the IoT device and the other for the BC Gateway. Moreover, the device must be registered on the BC together with a number of additional details such as ID, device name, device serial number, and a set of policies. When an IoT device is physically connected to the BC gateway, a link between the two smart contracts is created. Furthermore, if a user wants to access an IoT device, he/she can query the BC gateway’s smart contract to obtain the list of the devices connected to that gateway. He/she decides to accept or reject the privacy policies of the chosen device. If accepted, the user preferences are stored within the BC and the gateway and thus will be used in future when the user accesses the same IoT device via the same gateway. This type of access control system preserves the device policies and the users’ preferences from tampering and gives the whole environment a non-repudiation feature without a TTP.

It is, therefore, evident that one of the main objectives of companies is to merge the BC technology and IoT—in particular, to enable companies to share IoT data in a secure, efficient, and cost-efficient manner. IBM [64] developed a distributed infrastructure that perfectly matches the “IoT” by combining BC technology with BitTorrent and a messaging protocol called Telehash. The proposed system is called Adept, and it uses three different technologies to solve both technical and economic issues for the “IoT.” Brody et al. imagined a smart world where hundreds of billions of devices may, in the future, be connected to a single BC or a network of chains, communicating with each other through automated operations. To do that, there is the need to develop a platform that maintains intelligence at a device-level and can work without the manufacturer’s constant attention and maintenance. The reason to use BC technology, according to IBM, is the idea of how “this architecture could change business models for the IoT.” These architectures can create new business models to share, in any situation, not just data but computational power, bandwidth, or even electrical power through BC instructions. Moreover, IBM, in collaboration with 30 other banking and IT companies, wants to develop a BC of their own. It will be called Hyperledger [75].

Hyperledger is a software (San Francisco, CA, United States) that aims to create a scalable BC, allowing organizations to do business with anyone without the need for mutual trust. Hyperledger wants to go where BC has not yet arrived, by adding to the classic BC features new processes for more accurate verification of the identity of the involved people. Fabric, which was previously an IBM project called Open BC, became the first project incubated in Hyperledger and its name was changed to Fabric [76]. Fabric implements the technology of BC in a very flexible way, and, according to the authors, the same Bitcoin could be obtained using Fabric as a simple specialization. It can host contracts written in any development language. Three main architectural concepts are quite typical for any distributed ledger technology *(i) the ledger*: a universal register that contains the system status and transactions. It is organized as cryptographically linked blocks; *(ii) the transaction*: a request to perform a function on the ledger. The function is implemented by an executable code called chaincode (Smart Contract); *(iii) the chaincode*: code that implements a function that can alter the state of the system. The chaincode is also stored on the ledger.

Hyperledger BC counts three main modules, namely: *(i)* membership handling tasks about identity registration and management; *(ii)* BC and transactions that are the cores of the system. These modules manage all the BC services like consensus algorithms, distributed ledger, storage management, and communication p2p protocol (gRCP or Rest(deprecated)); and *(iii)* Chaincode that is the business logic. Chaincode is executed when transactions are submitted and validated. Hyperledger, furthermore, has different projects besides Fabric. Some of these are Sawtooth Lake [115] and Iroha [127]. In comparison to Fabric that *(i)* is written in GO, *(ii)* makes use of gRPC APIs and provides support for *(a)* Java Chaincode, *(b)* Chaintool, and *(c)* a client SDK for Node.js, Sawtooth Lake is written in Python and uses Rest APIs. The most interesting implementation proposed is the new consensus algorithm called PoET. Iroha is inspired by Fabric. It is written in C++ language and leverages on gRPC and Rest APIs as well. Another open-source framework focusing on IoT, which is having an impact on the cryptocurrency world, is IOTA [83]. IOTA is a cryptocurrency specified for micro-payments. IOTA is not based on the traditional BC technology, but it exploits TANGLE, which is a BC without blocks and miners. In other words, IOTA leverages on an acyclic graph. This solution makes the system highly scalable. Furthermore, IOTA is an asynchronous network that does not need mining, and thus it is a system with zero transaction fees. In case of conflict, a node can decide which transaction should be approved. Transactions are not approved in blocks but individually. Authors in [100] propose the first part of all the Hybrid-IoT platform. The platform consists of two parts: *(i)* generation of multiple PoW BCs, and *(ii)* creation of BFT framework to integrate BCs created in *(i)* so that they can interoperate. The sub-BCs are generated based on rules called sweet-spot guidelines. The sweet-spot guidelines enable the scaling of the BC. The flow of the transaction is as follows: transaction on PoW based sub-BCs are accepted after a PoW consensus. Inter sub-BC transactions are executed by the BFT inter-connector framework. After validation of transaction correctness and authenticity, the BFT inter-connector framework sends the transaction to target sub-BC for consensus. Moreover, by maintaining low latency in the transmission of interblockchain transactions, the BFT inter-connector framework allows the connection of a new sub-blockchain without deferring application execution. Chakraborty et al. in [101] propose a two-layered architecture to handle security to resource-constrained IoT nodes. The layer 0 consists of a node not capable of enforcing security primitives due to resource constraints while level N consists of primary and secondary nodes where primary nodes handle the processing and secondary nodes assist the primary nodes. The inability of layer 0 nodes to enforce security prevents them from communicating with other layer 0 nodes directly. It is possible only via other higher level nodes capable of enforcing security. This feature of preventing direct communication between resource-limited nodes is extended beyond layer 0.

Alphand et al. in [103] propose an IoT security management platform called IoTChain. It is composed of two components: *(i)* an authorization BC based on ACE framework, and *(ii)* the OSCAR [104] object security model modified with a group key scheme. The BC provides a flexible and trustless way to handle authorization while OSCAR uses the public ledger to set up multicast groups for authorized clients. English et al. in [106] demonstrate the contribution of BC technologies in realizing a robust Semantic Web, and how this robust Semantic Web can strengthen the BC in return. The authors propose a framework for improving the BC. The authors first demonstrate the model of a BC based URI naming scheme that positions the RDF data model. They then describe the composition of an extensible ontology for BCs. Korpela et al. in [107] investigate the requirements and functionalities of supply chain integration. Cloud integration can be expected to offer a cost-effective business model for interoperable digital supply chains. The authors explain how supply chain integration through the BC technology can achieve disruptive transformation in digital supply chains and networks.

Guan et al. in [105] try to address the side channel attack where an adversary may track the application usage patterns by analyzing the user’s electricity consumption profile or ambient light inside the house profile. The adversary may plan his/her attack based on these profiles. Authors propose a privacy-preserving and efficient data aggregation scheme. Private BC is used to store the data of members of a group. This preserves the privacy between groups. For privacy protection within groups, pseudonym approach (user with multiple accounts) is used. Authors have also proposed the use of bloom filters for fast authentication. Mettler in [108] reports the influence of BC on the pharmaceutical sector outlining the influences, goals, and potentials of BC. The author surveys various applications of BC in the health-care industry. BC can resolve problems like public health management, user-oriented medical research based on personal patient data as well as drug counterfeiting by eliminating the need for intermediaries. Ruta et al. in [109] propose a novel Service-Oriented Architecture (SOA) based on a semantic BC for registration, discovery, selection and payment. Such operations are implemented as smart contracts, allowing distributed execution and trust. The authors also reported experiments to assess the early evaluation of the proposal. The authors define a semantic resource/service discovery layer on a BC. The elementary tasks like registration, discovery are accomplished via Smart Contracts. The assets are semantically annotated and can be transferred by agents on the BC identified by their public keys. The semantic property of the transactions does not affect the consensus. The authors also integrate semantic matching for resource discovery.

## 6. Blockchain-Based Marketplaces

Over the next decade, with rapid deployment of IoT solutions, more than 75 billion devices will be connected to each other interacting in various manners [128]. The sheer volume of data generated by such devices and its influence on the future of our society will lead to substantial business opportunities worth tens of billions over the next few years. A significant obstacle to ‘Big Data’ vision is the creation of ‘Data Silos’ where a majority of data, generated by the smart environment, remains locked. Data silos are characterized by a closed environment with little to no sharing with outside. This often leads to 99% of unused data that, if allowed to flow freely, could potentially contain precious information [129]. A solution can be trading of data, leading to innovation for companies and generation of entirely new revenue streams of data that would otherwise remain unused. Thus, we will enter into an era of ‘Machine Economy’ where everything from sensor data to electricity and analytics to storage will be traded.

A data marketplace or data market is a pivotal element of the machine economy. Data marketplaces are online stores, typically offering data from different sources and markets. Usual data sources include advertising, personal information, business intelligence, research and market, and demographics. Data vendors may offer different data types like mixed or structured and may even provide data in customized formats to individual clients. Clients of such data sources can include institutions like market intelligence agencies, businesses, government, and analysts. In the following section, we discuss some of the data marketplaces based on BC. Table 9 summarizes the main aspects of significant data marketplace platforms.

Tangle, based on IOTA’s data marketplace, solves critical problems, namely: real-time micro-payment, and lack of ensured authenticity and provenance. Both the problems are addressed by making validation an intrinsic property of the network. This also results in a network being entirely decentralized in contrast to BC architectures which are centralized around mining-pools. Wibson is another decentralized data marketplace that solves the problem of control over personal information, financially incentivizing the personal information at the same time. Various principles of control over personal information, fairness, transparency, censorship resistance, and anonymity are at the core of Wibson.

The Raiblocks (XBR) project aims at monetizing the access to data services via APIs. The payments for the access are made using an XBR cryptographic token based on the Ethereum BC. Databroker DAO is a peer-to-peer data marketplace to buy and sell sensor data. Based on the Ethereum network, it uses smart contracts and its DTX tokens for a transaction. Its open-source Databroker DAO API handles access to the data and authorization and is called dAPI. Datum, based on smart contracts and BC, enables users to store structured data securely. Using the DAT token, it is also possible for the user to buy and sell the stored data. Datum achieves scalability by leveraging BigchainDB and IPFS. Weeve envisions the evolution of the IoT into the Economy of Things having an unfalsifiable, scalable and secure data marketplace supported by its platform. The marketplace enables IoT devices to index, process, and trade digital assets in a verifiable manner.

## 7. Challenges

When we talk about BC, we immediately think about Bitcoin. For this reason, we use Bitcoin as a reference example. It is clear that the problems that Bitcoin suffers from could be the same for the other Blockchain-based system. The first question is: what will happen if the number of nodes taking part in the BC network decrease? If no clients join the network, the Bitcoin network perishes, as the Bitcoin network is alive only if there are nodes online. Therefore, elimination of the Bitcoin network is possible in the case of a global blackout of the Internet. Thus, it may be inferred that it is a very improbable outcome that the Bitcoin network fails because, firstly, the Bitcoin as a cryptocurrency is having tremendous global success, thus people have no reason whatsoever to abandon the network, quite the opposite, given the incentives, and, secondly, a global and permanent Internet blackout, or even an extensive enough partition, has next to zero probability of becoming reality. However, the number of nodes in the Bitcoin network is decreasing, and the reason is located within technical aspects.

The first one is the size of the BC. This size is increasing day by day. Today, the whole BC weighs around 120 GB, and this makes hard to be a *full node* of the chain for users having a general purpose personal computer. Moreover, the Bitcoin block size is 1 MB (for security reasons), and thus, it can contain around 1700 transactions. Considering that the time to add a new block to the chain is on average 10 minutes, we have in the best case seven transactions per second [130]. This number is extremely little if compared to the VISA system (dozens of thousands). This little number of transactions per second can make the delay considerable (hours or days for a single payment). If these problems remain unsolved, this cryptocurrency will become useless. Adopted solutions are: *(i)* to reduce the size of each block by separating the data related to the digital signature, or *(ii)* to increase the block size thus increasing the number of transactions per block.

Although the latter solution may seem to solve the problem, it instead will introduce another one: the bigger the block size, the greater the computational power needed to mine a block. Therefore, the number of *full nodes* could continue to decrease consequently, negatively affecting the distributed nature of the BC system. For these reasons, private BCs are catching on. Instead of having a public and uncontrolled network, it is possible to create a system where the access permissions are strictly controlled. In such a network, only some users have the right to read or modify the chain. The basic idea of a fully decentralized system fails in this case, especially if these private chains are controlled by big companies or banks. This is not only a financial problem, and it goes beyond the Bitcoin system.

The smart contracts open new futuristic perspectives: in the future, an individual may decide to rent a car or a house using cryptocurrency like Bitcoin or others such as Litecoin and Ethereum. Assuming that the tenant and individual that wants to rent the asset (for example a home) stipulated a smart contract previously, the individual could send “coins” to the smart contract that in turn enables the individual’s private crypto-key to unlock the door for a period specified within the contract. If the individual does not send coins periodically to the smart contract, this can automatically remove the validity of the crypto-key, disabling user access to the house’s door.

With the increase in tokens, investors, users, and other entities, BC is facing the issue of scalability. Scalability, as a challenge, can be observed concerning data size, response time, and cost. Rapid adoption of BC technology increased the number of transactions. With a gas limit on the block, the number of transactions that can be included in the block is limited. With limited block size, the wait time for the addition of transaction to the BC increases. In addition, to handle the increased traffic caused by more users and more transactions, more nodes are needed to process them, thereby increasing the cost. Various techniques are proposed to address the problem of scalability like Segwit, block size increase, Sharding, PoS, off chain state channels, and Plasma. A lot of research still needs to be done to propose a comprehensive solution.

In light of the above, are we sure that these technologies and their features are the panaceas for every problem? We must be careful in managing the power of the technology because what we think to be utopia could easily become dystopia and the boundary between them is very thin. If on the one hand, the smart contract automatizes, in a secure manner, transactions among individuals, on the other hand, it could be a very strict instrument that removing the third party entity eliminates the only entity able to protect human rights. This is because everything is managed automatically with the help of smart contracts and autonomously interacting with the IoT. Therefore, this technology, born free from the trust in central entities, could make companies, banks or governments stronger than in the past, ignoring actors that could take care of the citizen’s rights. Thus, a decentralization technology could be transformed into a means to exert centralized control nonetheless.

## 8. Conclusions

An IoT ecosystem has numerous vulnerabilities concerning confidentiality, privacy, and data integrity. For this reason, the researchers and developers of the ICT sector decided to integrate “security by design” technology within an environment such that IoT overcomes the limitations. BC, being one such technology, grants authenticity, non-repudiation, and integrity by default, and utilizing smart contracts, manages authorization and automation of transactions as well. In our survey, we (i) considered different application areas, organizing the available literature in these fields, (ii) presented two usage patterns that are: device manipulation and data management (open marketplace solution), and (iii) discussed the development level of some of the presented solutions.

In our opinion, there are many possibilities for future research paths. We believe that, in an era characterized by the pervasive use of smart devices and production of the enormous amount of data (Big data), the primary two necessities are: *(i)* development of a solution to grant data privacy and integrity; and *(ii)* design of system able to manage the unique identity of devices in a tamper-proof manner. As per the paper scope distribution, we see that the research in the direction of IoT and BC is in an early phase. A lot of research needs to be done in specific domains like Smart Energy and Smart Manufacturing. Being in the initial phase, very little research is done with addressing the problem of scalability of the BC solution. Research is being carried out regarding consensus protocols to enable the scalability of the integration. From the distribution, we can see that a lot of research has been carried out in the field of smart homes and smart cities. The next logical step lies in breaking down the most widely adopted platforms in self-contained subsystems, and then building full-featured stacks from standardized/pluggable components. In addition, protection against innovative attacks like side channel analysis can be an interesting venture. Another interesting research direction can be the application of BC to solve the problem of data exchange and trading. With the ubiquity of IoT devices and increasing production of data, attempts have started to monetize the data giving birth to the Machine Economy. BC can streamline the negotiation processes, eliminating the need for a trusted intermediary.

## Figures and Tables

**Table 1:** Main survey papers categorized by contribution.

Contribution	Papers
Challenges & Existing platforms	[5,8], this paper
Security Challenges in IoT and BC as Solution	[6,12], this paper
Internet of Things (IoT) Security and Stalker Attack	[7], this paper (partially)
Consensus (mechanisms) Taxonomy	[9], this paper

**Table 2:** Consensus Protocol Comparison.

	PoW	PoS	PoET	BFT and Variants	Federated BFT
*(i)* BC type	Permissionless	Both	Both	Permissioned	Permissionless
*(ii)* Transaction finality	Probabilistic	Probabilistic	Probabilistic	Immediate	Immediate
*(iii)* Transaction rate	Low	High	Medium	High	High
*(iv)* Token needed?	Yes	Yes	No	No	No
*(v)* Cost of participation	Yes	Yes	No	No	No
*(vi)* Scalability of peer network	High	High	High	Low	High
*(vii)* Trust model	Untrusted	Untrusted	Untrusted	Semi-trusted	Semi-trusted

**Table 3:** Papers’ scope of application. GA: Generic Application, SM: Smart Property, SH: Smart Home, SC: Smart City, SE: Smart Energy, SM: Smart Manufacturing, and DM: Data Marketplace.

Papers	GA	SP	SH	SC	SE	SM	DM
Sun et al. [3]	-	-	-	✓	-	-	-
TransActive Grid [10]	-	-	-	-	✓	-	-
Filament [59]	✓	-	-	-	-	-	-
Jentzsch et al. Slock.it [13]	-	-	✓	-	-	-	-
Zhumabekuly Aitzhan et al. PriWatt [60]	-	✓	-	✓	✓	-	-
Axon et al. [61]	✓	-	✓	-	-	-	-
Bahga et al. [62]	✓	-	-	✓	-	✓	-
Biswas et al. [63]	✓	-	-	✓	-	-	-
Brody [64]	✓	-	✓	✓	-	-	-
Cha et al. [65]	✓	-	-	-	-	✓	-
Conoscenti et al. [66]	-	-	✓	✓	-	-	-
Dorri et al. [67,68]	✓	-	✓	-	-	-	-
Fromknecht et al. [69]	✓	-	-	-	-	-	-
Ghuli et al. [70]	-	✓	-	-	-	-	-
Hardjono et al. [71]	-	-	-	✓	-	✓	-
Hashemi et al. [72]	-	-	-	✓	-	-	-
Herbert et al. [73]	-	✓	-	-	-	-	-
Huh et al. [74]	-	-	✓	-	-	-	-
IBM Hyperledger [75,76]	✓	-	-	-	-	-	-
Leiding et al. [77]	✓	-	-	-	-	-	-
Lombardi et al. [78]	-	-	✓	✓	✓	-	-
Munsing et al. [79]	-	-	-	✓	✓	-	-
Nehaï et al. [80]	-	-	✓	✓	✓	-	-
Ouaddah et al. [81]	✓	-	-	-	-	-	-
Popov [82] IOTA [83]	✓	-	-	-	-	-	-
Prabhu et al. [84]	✓	-	-	-	-	-	-
Shafagh et al. [85]	✓	-	-	-	-	-	-
Sikorski et al. [86]	-	-	-	-	-	✓	-
Wilkinson et al. Storj [87]	-	-	✓	-	-	-	-
Wörner et al. [88]	✓	-	-	-	-	-	-
Xu et al. Sapphire [89]	✓	-	-	-	-	-	-
Zhang et al. [90]	-	✓	-	-	-	-	-
Zyskind et al. [91]	✓	-	-	-	-	-	-
Ralph Deters [92]	-	-	-	-	-	-	-
Gorilovsky et al. (Moeco) [93]	-	-	-	-	-	-	-
Schiener [94]	✓	-	-	-	-	-	✓
Wibson [95]	✓	-	-	-	-	-	✓
XBR [96]	✓	-	-	-	-	-	✓
Niekerk et al. [97]	✓	-	-	-	-	-	✓
Haenni [98]	✓	-	-	-	-	-	✓
Davidsen et al. [99]	✓	-	-	-	-	-	✓
Sagirlar et al. [100]	✓	-	-	-	-	-	-
Chakraborty et al. [101]	✓	-	-	-	-	-	-
Wu et al. [102]	-	-	✓	-	-	-	-
Alphand et al. [103]	✓	-	-	-	-	-	-
Vučinić et al. [104]	✓	-	-	-	-	-	-
Guan et al. [105]	✓	-	-	-	✓	-	-
English [106]	✓	-	-	-	-	-	-
Korpela et al. [107]	✓	-	-	-	-	✓	-
Mettler [108]	✓	-	-	-	-	-	-
Ruta [109]	✓	-	-	-	-	-	-

**Table 4:** Usage Pattern Organization.

Papers	Device Manipulation	Data Manipulation
TransActive Grid [10]	-	✓
Filament [59]	-	✓
Jentzsch et al. (Slock.it) [13]	✓	-
Zhumabekuly Aitzhan et al. (PriWatt) [60]	-	✓
Axon et al. [61]	✓	-
Bahga et al. [62]	✓	-
Biswas et al. [63]	-	✓
Brody [64] (ADEPT)	✓	-
Cha et al. [65]	✓	✓
Conoscenti et al. [66]	-	✓
Dorri et al. [67,68]		✓
Ghuli et al. [70]	✓	-
Hardjono et al. [71]	✓	✓
Hashemi et al. [72]	-	✓
Herbert et al. [73]	✓	-
Huh et al. [74]	✓	-
Hyper-Fabric [76]	-	✓
Leiding et al. [77]	-	-
Lombardi et al. [78]	✓	-
Munsing et al. [79]	✓	-
Nehaï et al. [80]	-	✓
Ouaddah et al. [81]	✓	✓
Prabhu et al. [84]	✓	✓
Shafagh et al. [85]	-	✓
Sikorski et al. [86]	-	✓
Wilkinson et al. (Storj) [87]	✓	✓
Wörner et al. [88]	✓	✓
Zhang et al. [90]	-	✓
Zyskind et al. [91]	-	✓
Xu et al. (Sapphire) [89]	✓	-
Ralph Deters [92]	-	✓
Gorilovsky et al. (Moeco) [93]	-	✓

**Table 5:** System’s Development Level.

Solutions	Theory	Simulation	Prototype	Pre-Product	Product
TransActive Grid [10]	-	-	-	-	✓
Filament [59]	-	-	-	-	✓
Slock.it [13]	-	-	-	-	✓
PriWatt [60]	✓	-	-	-	-
BPIIoT [62]	-	-	✓	-	-
IBM Adept [64]	✓	-	-	-	-
Cha et al. [65]	-	-	✓	-	-
Dorri et al. [67,68]	-	-	✓	-	-
CertCoin [69,110]	-	-	✓	-	-
ChainAnchor [71]	-	-	✓	-	-
Hashemi et al. [72]	-	-	✓	-	-
Huh et al. [74]	✓	-	-	-	-
IBM Hyperledger [75,76]	-	-	-	✓	-
IOTA (TANGLE) [82,83]	-	-	-	✓	-
Sikorski et al. [86]	✓	-	-	-	-
Storj [87]	-	-	-	✓	-
Sapphire [89]	-	-	✓	-	-
Enigma [91,111]	-	-	-	-	✓
Moeco [93]	-	-	-	-	✓

**Table 6:** Challenges Addressed by the Solution.

**Confidentiality**	PriWatt [60]; Axon et al. [61]; Brody [64]; Cha et al. [65]
	Dorri et al. [67,68]; Hardjono et al. [71]; IBM Hyperledger [75,76];
	Munsing et al. [79]; Lombardi et al. [78]; Nehaï et al. [80];
	Ouaddah et al. [81]; Shafagh et al. [85]; Storj [87]; Enigma [91,111]
	Filament [59]; Vučinić et al. [104]; Guan et al. [105];
	Mettler [108]; Haenni [98]; Davidsen et al. [99];
	Schiener [94]; Wibson [95]; Niekerk et al. [97];
**Authentication/**	Filament [59]; PriWatt [60]; Axon et al. [61]; Brody [64];
**Id-Management**	Slock.it [13]; Cha et al. [65]; CertCoin [69]; Ghuli et al. [70];
	Hardjono et al. [71]; Hashemi et al. [72]; Huh et al. [74];
	AuthCoin [77]; IBM Hyperledger [75,76]; Ouaddah et al. [81];
	Shafagh et al. [85]; Enigma [91,111]; Wu et al. [102]; English [106]
	Vučinić et al. [104]; Mettler [108]; Haenni [98]; Davidsen et al. [99];
	Schiener [94]; Wibson [95]; Niekerk et al. [97];
**Integrity**	Biswas et al. [63]; Dorri et al. [67,68]; IBM Hyperledger [75,76];
	Lombardi et al. [78]; Ouaddah et al. [81]; Shafagh et al. [85]; Storj [87];
	Xu et al. Sapphire [89]; Enigma [91,111]; IOTA (TANGLE) [82,83]
	Mettler [108]; Haenni [98]; Davidsen et al. [99]; Chakraborty et al. [101];
	Schiener [94]; Wibson [95]; Niekerk et al. [97]; Sagirlar et al. [100];
**Availability**	Bahga et al. [62]; Brody [64]; Dorri et al. [67,68]; IBM Hyperledger [75,76];
	Ruta [109]; Lombardi et al. [78]; Storj [87]; Enigma [91,111];
	Mettler [108]; Haenni [98]; Davidsen et al. [99];
	Schiener [94]; Wibson [95]; Niekerk et al. [97];
**Non-repudiation**	TransActive Grid [10]; Slock.it [13]; PriWatt [60]; Axon et al. [61];
	Bahga et al. [62]; Herbert et al. [73]; IBM Hyperledger [75,76];
	Nehaï et al. [80]; Zhang et al. [90]; English [106]; Mettler [108];
	Haenni [98]; Davidsen et al. [99]; Schiener [94]; Wibson [95];
	Niekerk et al. [97];

**Table 7:** Use Cases of the Analyzed Solutions.

Category	Sub-Category	By Means of	Papers
Transaction or	Energy	*(i)* Transactive (a distributed consensus system	[10]
Sharing		for Microgrids) and Ethereum-based BC	
Systems		to regulate energy transactions.	
		*(ii)* Token-based BC for Bitcoin payments,	PriWatt [60]
		ECDSA-based multi-signature approach	
		and anonymous encrypted messages	
		(Bitmessage [112]).	
		*(iii)* Smart contracts deployed on permissioned	[78]
		BC. Each node (ETSE module), interacting with	
		smart meters and other nodes, stores energy	
		transactions on BC.	
		*(iv)* Distribution of energy through a decentralized	[79]
		algorithm by implementing Alternating Direction	
		Method of Multipliers (ADMM) on an	
		Ethereum-based BC.	
		*(v)* A merging of the Smart grid model with the	[80]
		ElectricChain [113] and smart contracts	
		to manage strategies and consumption patterns.	
		*(vi)* BC storing and publishing energy offers and	[86]
		energy transactions. As future work, conjunction	
		of the J-Park Simulator (JPS) is planned.	
	Data	*(i)* M2M transactions based on	[62]
		smart contract exploitation.	
		*(ii)* A merging of BC, BitTorrent and Telehash	ADEPT [64]
		protocol.	
		*(iii)* Types of BC: Smart Home and	[67,68]
		Overlay network BCs. Cloud Storage to save	
		smart home devices’ data.	
		*(iv)* BC storing transactions about device	Chain-
		commissioning.	Anchor [71]
		*(v)* Three layers system. Tokens-based BC	[72]
		system.	
		*(vi)* A customized token-based BC to manage	[73]
		software validation.	
		*(vii)* Flexible permissioned smart contract-	Hyper-
		based BC.	Fabric [76]
		*(viii)* Distributed Ledger implemented by a	IOTA [83]
		tangle or directed acyclic graph (DAG).	
		*(ix)* BC and Distributed File System (DFS) (e.g.,	[89]
		Hadoop Distributed File System (HDFS)) as	
		Object storage system.	
		*(x)* Token-based BC to manage data	[90]
		ownership transactions.	
		*(xi)* BC, DHT and other communication	Enigma [91],
		protocol to develop data storage system.	[85],
			Storj [87]
		*(xii)* Ethereum token-based BC to store	Moeco [93]
		data transfers and payments.	
	Goods	*(i)* BC, Telehash protocol and radio hardware	[59]
		to create an interaction layer between devices.	
		*(ii)* Monitoring real-time transactions to read	[67,68]
		real-time device’s data.	
		*(iii)* Smart objects and buyers and sellers able	[90]
		to transact with the BC (Smart Property).	
Ownership	Data	*(i)* Enhanced Privacy ID (EPID) protocol	Chain-Anchor [71]
		& BC as anonymous device commissioning	
		and decommissioning register.	
		*(ii)* Master Bitcoin Model the pair “Vendor-	[73]
		PubKey/Bitcoin” as proof of ownership.	
		*(iii)* ColoredCoin/Tokens (Smart Property).	[90]
	Goods	*(i)* CIA	[70]
		(to get a private & public key pair) +	
		BC transaction payments.	
		*(ii)* Smart contracts and tokens.	[90]
Identity	PKI-Based BC	*(i)* Separation of the identity value from a	[61]
Management		series of short-term public keys	
		posted to a BC.	
		*(ii)* Built-on Namecoin BC where identities	Certcoin [69,110]
		and public keys are posted in pairs. Use of	
		Cryptographic accumulators &	
		DHTs [114].	
		*(iii)* Combination of a decentralized WoT,	Authcoin [77]
		BC and bidirectional Challenge-response	
		validation and authentication process	
		of public keys.	
	BC-Based	*(i)* The anonymous identity protocol	Chain-Anchor [71]
		RSA-based EPID and DAA schemes.	
		*(ii)* Implicit authentication by adding of	[74]
		allowed signature and public key	
		within smart contracts.	
		*(iii)* Membership modules manage	Hyper-Fabric [76]
		registration and authentication tasks.	
		*(iv)* BC as a backbone; Device to	[84]
		Device authentication.	
		*(v)* An off-chain technology (DHT)	Enigma [91]
		linked to a BC.	
Access Control	Data	*(i)* Data owners issue the tokens to	[72]
		regulate the data access.	
		*(ii)* Data Broker and Provenance Verifier	Chain-Anchor [71]
		(DB-PV), semi-permissioned BC and	
		ChainAnchor consensus nodes.	
		*(iii)* A local home BC. An access control	[67,68]
		list stored into a policy header of the BC.	
		*(iv)* Tokens (IoTCoins) bought with Bitcoins.	[90]
		IoTCoins represent the data access. The seller	
		encrypts data with the buyer public key.	
		*(v)* FairAccess model based on smart contract to	[81]
		define policies & authorization decisions.	
		*(vi)* Smart contracts stored in external BC.	Enigma [91]
		*(vii)* Each block of the BC contains data	[85]
		for access permissions.	
		*(viii)* Announcement or Smart Contracts	[92]
		approaches.	
	Device	*(i)* Ethereum-based BC , smart contracts and	Slock.it [13]
		physical “Slock” devices.	
		*(ii)* JSON-based privacy policies and smart	[65]
		contracts for the devices registered within the BC.	
Other		*(i)* A BC-based communication layer and networking	[63]
		protocols (Ethereum, NXT, Telehash).	
		*(ii)* BC-based routing system.	Moeco [93]

**Table 8:** Consensus Algorithms.

Papers	Consensus Algorithm
Sun et al. [3]	not specified
Filament [59]	PoElapsedTime (Fabric Sawtooth platform [115])
Slock.it [13]	Ethereum-like Ethash
Zhumabekuly Aitzhan et al. [60]	Bitcoin-like PoW
Axon et al. [61]	Bitcoin-like PoW
Bahga et al. [62]	Ethereum-like
Biswas et al. [63]	Ethereum-like
Brody [64]	Ethereum-like
Cha et al. [65]	Ethereum-like
Conoscenti et al. [66]	not specified
Dorri et al. [67,68]	no PoW
Fromknecht et al. [69]	Bitcoin-like PoW (CertCoin/NameCoin platform)
Ghuli et al. [70]	Bitcoin-like PoW
Hardjono et al. [71]	Bitcoin-like PoW
Hasheme et al. [72]	Bitcoin-like PoW
Herbert et al. [73]	Bitcoin-like PoW
Huh et al. [74]	Ethereum-like
IBM Hyperledger [75,76]	PBFT
Leiding et al. [77]	depending on the underlying BC platform
Lombardi et al. [78]	Ethereum-like
Munsing et al. [79]	Ethereum-like
Nehaï et al. [80]	PoW/PoStakeTime (Solarcoin)
Ouaddah et al. [81]	not specified
IOTA (TANGLE) [83]	no mining process
Prabhu et al. [84]	Bitcoin-like
Shafagh et al. [85]	not specified
Sikorski et al. [86]	Round Robin-based
Wilkinson et al. [87]	PoStorage / PoRedundancy
Wörner et al. [88]	Bitcoin-like
Xu et al. [89]	Ethereum-like
Zhang et al. [90]	depending on the underlying BC platform
Zyskind et al. (Enigma) [91]	Parent layer consensus
Deters [92]	not specified
Gorilovsky et al. Moeco [93]	Exonum [116] custom-built BFT (no mining)

**Table 9:** Data Marketplace Platforms.

Platform	Public Ledger	Available
IOTA’s data marketplace [94]	Tangle	Real time
		Proof-of-Concept
Wibson’s data marketplace [95]	Ethereum	Alpha version app
Project XBR [96]	Ethereum	Available
Databroker DAO [97]	Ethereum	Beta-version
Datum [98]	Ethereum	Beta-version
Weeve [99]	IOTA, Ethereum, Hyperledger	Beta-version
